# Value of CIM, CLO Test and Multiplex PCR for the Diagnosis of *Helicobacter Pylori* Infection Status in Patients with Gastritis and Gastric Ulcer

**DOI:** 10.31557/APJCP.2019.20.11.3497

**Published:** 2019

**Authors:** Tran Thien Trung, Tran Anh Minh, Nguyen Tuan Anh

**Affiliations:** 1 *Department of Surgery, Faculty of Medicine, University of Medicine and Pharmacy, *; 2 *Molecular Biomedical Center, University Medical Center Branch No. 2, University of Medicine and Pharmacy, *; 3 *Research Center for Genetics and Reproductive Health, School of Medicine, Vietnam National University, Ho Chi Minh City, Vietnam. *

**Keywords:** CIM test, CLO test, Helicobacter pylori, multiplex PCR, vietnamese

## Abstract

**Objective:**

To assess the value of Current Infection Marker (CIM) test, Campylobacter-Like Organism (CLO) test, and the multiplex polymerase chain reaction test (PCR) for the diagnosis of *Helicobacter pylori* (*H. pylori*) infection in a Vietnamese population.

**Methods:**

Targeted suitable patients were recruited. CIM test, CLO test and multiplex PCR were used to diagnose for *H. pylori *infection. Patients were considered positive for *H. pylori *when at least two of the three tests were positive. The performance of each of the three tests was compared to the *H. pylori *positive populations as defined.

**Result:**

Amongst 201 patients with a mean age of 40.5 (range, 18-74) years, there were 115 females and 86 males. Of the 201 patients, 107 (53.2%) were diagnosed as *H. pylori *positive according to the defined criteria. The positive patients obtained with CLO test, CIM test and multiplex PCR were 38.3%, 59.2% and 72.1%, correspondingly. The full performance of the three tests as highlighted in order as above were 85.07%, 83.08% and 81.09%, respectively. The positive rate of CLO test was the lowest, with 38.3% positive, but this method was the most accurate, with the accuracy of 85.07%. This suggested that CLO test has the highest specificity among the three. The sensitivity, specificity, positive, negative predictive values and accuracy of the CLO / CIM / multiplex PCR tests were 71.96% / 89.72% / 100%, 100% / 75.53% / 59.57%, 100% / 80.67% / 73.79%, 75.81% / 86.59% / 100%, and 85.07% / 83.08% / 81.09%, respectively.

**Conclusion:**

All the three methods have high accuracy for the diagnosis of *H. pylori *infection in the Vietnamese population with gastritis and gastric ulcers. These tests can be employed in the clinical settings for the Vietnamese population. CLO test should be used in combination with the other tests to reduce false-negative results.

## Introduction

*H. pylori *is a bacterium involving directly in the pathogenesis of various gastroduodenal diseases, especially gastric cancer (Pellicano et al., 2016). The prevalence of *H. pylori *infection varies across countries worldwide. The prevalence of *H. pylori *infection is about 25-50% in developed countries, but more than 80% in the developing ones (Hosseini et al., 2012). In Vietnam, the prevalence of *H. pylori *infection ranges from 65.6% to more than 74.6% (Fock and Ang, 2010; Nguyen et al., 2010), particularly 78.8% in Hanoi (Hoang et al., 2005) and 80.5% in Ho Chi Minh city (Binh et al., 2017). 

It is proven that *H. pylori *infection is one of the etiology of conditions such as gastritis, peptic ulcer and gastric cancer. Therefore, accurate clinical diagnosis for the treatment of *H. pylori *infection is crucial for the infected population.

Clinicians are now facing difficulties in the clinical diagnosis of *H. pylori*. Many available diagnostic tests have shown questionable value and accuracy in the diagnosis of the *H. pylori *infection. This confounded the health practioners in making the decision for treatment.

Nowadays, there are different methods for *H. pylori *detection. They are generally divided into two groups: invasive and non-invasive diagnostic tests. The invasive testing requires the use of gastric biopsies obtained by endoscopic examination, such as CLO test, bacterial culture, histology and molecular methods (PCR). The non-invasive testing includes stool antigen test, serology test and urea breath test (Versalovic, 2003). Each method has its own advantages and disadvantages.

In Vietnam, endoscopic examination coupled with CLO test, or more recently, PCR testing, is used to diagnose *H. pylori *infection. The sensitivity and specificity of multiplex PCR comparing with serology are 96.95% and 86.17% respectively while CLO test has high false-negative ratio and should be reevaluated (Trung et al., 2013). Suspected antimicrobial resistance cases and related cancer patients are further tested through bacteria culture testing and histology. Non-invasive tests such as urea breath test and serology are also commonly used. Serology tests are often used in diagnosis and epidemiological studies, but not for *H. pylori *eradication therapy selection and monitoring. However, CIM test, based on the detection of H. pylori-specific antibiotics, is a simple, rapid, and reliable test with the sensitivity and specificity of 85.7% and 96.9% correspondingly when the CIM test was used 6 months after the end of anti-*H. pylori *therapy, using urea breath test (UBT) as a gold standard (Wang et al., 2008). Though, UBT is an expensive method compared to the others. In general, the non-invasive *H. pylori *detection methods are gaining popularity every passing day for their advantages (Schabereiter-Gurtner et al., 2004).

According to the European guidelines, at least two positive test results from different testing methods are required for the confirmation of *H. pylori *infection in a patient (Bah et al., 1995). A recent study suggested that the gold standard for determining *H. pylori *infection status may be nested PCR thanks to its relatively higher sensitivity and specificity (Patel et al., 2014). However, the suitable diagnostic method for *H. pylori *infection status still depends on the prevalence and divergence of *H. pylori *strains, the availability of testing methods and patients’ clinical conditions (Tongtawee et al., 2016). Besides, successful *H. pylori *eradication after treatment could be determined by urea breath test, stool antigen test and PCR (Tongtawee et al., 2016).

To determine values of diagnostic methods for use in Vietnamese medical practice, we evaluated three methods including CIM test, CLO test and multiplex PCR in patients with gastritis. The purpose of this study is to determine the accuracies of these methods, either individually or in combination, for *H. pylori *detection at University Medical Center Ho Chi Minh City, Branch Number 2.

## Materials and Methods

*Patient population*

A cross-sectional study was carried out from January 2016 to December 2016 at University Medical Center Ho Chi Minh City, Branch Number 2. Patients were recruited volunteering for gastrointestinal disease examination. 

The recruitment criteria included the following: patients who must be from Ho Chi Minh City and southern provinces, at least 16 years old, with symptoms such as epigastric pain, flatulence, belching, indigestion, or fresh stool disorder; who underwent upper gastrointestinal endoscopy and were diagnosed as gastritis or gastric ulcer, and received no pharmacological intervention relating to gastric diseases, such as antimicrobials, one month before. Patients were tested by all three methods, comprising gastric endoscopy plus CLO test, CIM test and multiplex PCR. The exclusion criteria included patients with gastric cancer and/or patients taking any type of drugs and antimicrobials at the point of time participating in the study or patients that had taken drugs up to a month prior.

*Gastric biopsies*

Two biopsies were taken from the antrum of the greater and lesser curvature from each patient through upper gastrointestinal endoscopy (Uotani and Graham, 2015). These samples were used to detect *H. pylori *infection by CLO test and multiplex PCR. Serum samples from these patients were also collected to test for *H. pylori *antibodies by CIM test. All samples were stored at 4^o^C until further processing within a day.

*In-house CLO test*

CLO test was performed by putting the biopsies into a solid supporting medium containing urea and phenol red as a pH indicator. The testing media components are as followed: 2 g Urea, 2.5 ml aqueous phenol red 0.4% wt/vol, 0.14 g NaH_2_PO_4_.H_2_O 10 mmol, distilled water to 100 ml (pH 6.3-6.5), 0.4 g agar (Merck). All chemicals were prepared and used within a month. Reference strains (*Helicobacter pylori *J99 for positive-CLO test and clinical *E. coli *for negative-CLO test) were used to check the CLO test before using. Urease enzyme produced by the bacteria hydrolyses urea to release CO_2_ and NH_3_. The release of ammonia increases the pH of the test medium and changes the colour of the pH indicator from yellow to pinkish or red. The colour change indicates the biopsy specimens are positive to *H. pylori*.

*CIM test*

For CIM test, Assure® *H. pylori *Rapid Test kit from MP Biomedicals Asia Pacific Pte Ltd (Singapore) was used to diagnose *H. pylori.* CIM is an *H. pylori*-specific novel recombinant protein identified from the cDNA library. CIM test is an indirect solid-phase immunochromatographic assay with specific antigens to detect IgG antibodies that are produced by active *H. pylori *infection and present in the blood sample. The usefulness of the CIM test is easy-to-use, non-invasive, and might detect current *H. pylori *infection as shown in some studies (Hung et al., 2002) even though it needs more than 6 months after anti-*H. pylori *therapy to differentiate the past or current infection correctly (Wang et al., 2008). To ensure accurate results were produced by the CIM test regarding the *H. pylori *infection, every step in the protocol was performed according to the manufacturer’s guidelines. Current *H. pylori *infection was determined only if the Control Line, Test Line and CIM line were present together (Wang et al., 2008). 

*Multiplex PCR*

DNA was extracted from biopsies using Qiacube automated purification system and kit (Qiagen) according to the manufacturer’s instructions. Optimisation of the multiplex PCR components and conditions were performed to determine the optimum conditions for the detection of specific fragments of *cagA* and *vacA* genes of the bacteria in PCR detection system (Bio-Rad). Four primer pairs were used in one reaction. Sequences of primers used were as previously described. The primer sequences are showed in Table 3. Concentration of primers (200 nM for each *cagA* (Park et al., 2003), *vacA *s1/s2 (Park et al., 2003; Kumar et al., 2008), *vacA* m1/m2 (Park et al., 2003; Kumar et al., 2008) primers and 50 nM for SMAD4 primers as internal control), MgCl_2_ (2 mM), h-Taq DNA polymerase (1 unit), dNTPs mix (200 µM), 1x PCR buffer and 5 µl extracted DNA were used in a total 25µl reaction volume. The multiplex PCR was used to detect *H. pylori *and its genotypes, such as *cagA* status (349 bp) and *vacA* genotypes (s1/s2 and m1/m2 with the fragments of 259/286 and 567/642 bp, correspondingly). The optimum amplification conditions were achieved through an initial denaturation of target DNA at 95^o^C for 15 minutes, followed by 40 cycles of denaturation at 95^o^C for 20 seconds, annealing at 60^o^C for 40 seconds and extension at 72^o^C for 40 seconds. The final cycle included extension for 6 min at 72^o^C. Ten microliter of multiplex PCR products were subjected to electrophoresis on 5% (wt/vol) agarose gel in 120 mA for 40 min using submarine horizontal electrophoresis apparatus. The gels were stained with EcoDye solution (Biofact) and PCR bands were visualized under ultraviolet light with 100 bp DNA ladder (Solgent). The reference *H. pylori *strains, such as ATCC 700824 (J99 strain) and ATCC 51932 (Tx30a), were used as positive controls. The presence of *H. pylori *in gastric biopsy samples by multiplex PCR was determined positive when there was at least one of the following specific products, including 349 bp (*cagA*), 259 bp (*vacA* s1), 286 bp (*vacA* s2), 567 bp (*vacA* m1), and 642 (*vacA *m2) fragments showing on gel (Anh et al., 2010).

*Definition of H. pylori infection*

The definition of *H. pylori *infection in this study required at least two positive tests of the three tests, comprising of CLO test, CIM test and multiplex PCR. The gold standard to measure the sensitivity, specificity, positive and negative predictive values and accuracy of the three tests in this study was based on the above definition of *H. pylori *infection status according to the European guidelines.

*Statistical analysis*

Statistical analysis was performed using the Statistical Package for the Social Science (SPSS) version 20.0. Descriptive statistical analysis was used to describe the characteristics of patients’ age and gender. Pearson’s Chi-square test was used to evaluate the *H. pylori *diagnostic rates by different methods. P value less than 0.05 was considered significant. Kappa (κ) measures were used to evaluate the agreement between diagnostic results produced by three methods with the *H. pylori *infection status. The strength of agreement was considered to be slight if κ ≤ 0.2; fair if 0.2 < κ ≤ 0.4; moderate if 0.4 < κ ≤ 0.6; substantial if 0.6 ≤ κ ≤ 0.8 and almost perfect if κ > 0.8 (Landis and Koch, 1977). Analysis to determine the sensitivity, specificity, positive and negative predictive values, accuracy of the tests was calculated by Med Calc program (http://www.medcalc.org/calc/diagnostic_test.php). 

## Results

There were 201 qualified patients recruited in this study. All patients were of Vietnamese nationality. Details in the demographic characteristics of the patients are indicated in Table 1.

The mean age of the patients was 40.5±11.6 (95% CI: 38.9-42.2) years old, ranging from 18 to 74. The age followed the standard distribution (p=0.362; Skewness Test). There were 42.8% (86/201; 95% CI: 36.8-50.7) male and 57.2% (115/201; 95% CI: 49.3-63.2) female. The majority of patients were diagnosed with gastritis (96.0%; 193/201); only a small percentage was diagnosed with gastric ulcer (4.0%; 8/201).

*H. pylori infection status diagnosed by CLO test, CIM test and multiplex PCR*

There was a significantly statistical difference between each test type and *H. pylori *infection status (p < 0.001). The sensitivity, specificity, positive and negative predictive values and accuracy of the CLO test were 71.96% (77/107), 100% (94/94), 100% (77/77), 75.81% (94/124), and 85.07% (171/201), respectively (Table 2). The agreement of CLO test to the *H. pylori *infection status was substantial with κ value equal to 0.706±0.047.

The sensitivity, specificity, positive and negative predictive values and accuracy of the CIM test were 89.72% (96/107), 75.53% (71/94), 80.67% (96/119), 86.59% (71/82) and 83.08% (167/201), respectively (Table 2). The agreement of CIM test to the *H. pylori *infection status was also substantial with κ value equal to 0.658±0.053.

The sensitivity, specificity, positive and negative predictive values and accuracy of the multiplex PCR were 100% (107/107), 59.57% (56/94), 73.79% (107/145), 100% (56/56), and 81.09% (163/201), respectively (Table 2). The agreement of multiplex PCR to the *H. pylori *infection status was substantial with κ value equal to 0.611±0.052.

The proportion of positive patients was different depending on the testing method. The lowest number of positive patients was obtained with CLO test (38.3%). Multiplex PCR gave the highest number of positive patients (72.1%). CIM has produced the average number of positive patients (59.2%), compared to the other two methods. There were significant statistical differences in *H. pylori *diagnosis between each test in pair, such as CIM / CLO test, CLO test / multiplex PCR, and CIM / multiplex PCR (p ≤ 0.001).

When combining three testing methods for *H. pylori *diagnosis, the number of negative patients for all three methods was 16.4% (33/201). The number of positive patients with at least one method was 83.6% (168/201). The number of positive patients with at least two methods was 53.2% (107/201) and with all three methods was 33.3% (67/201). 

All patients with positive CLO test (38.3%; 77/201) also were positive with multiplex PCR while their CIM tests were negative (14.3%; 11/77) or positive (85.7%; 66/77). In negative CLO test results (61.7%; 124/201), there were 42.7% (53/124) patients positive and 57.3% (71/124) patients negative to CIM test. Results of multiplex PCR could be negative or positive in these cases. For cases with positive CIM test, 24.2% (30/124) was positive and 18.6% (23/124) was negative with multiplex PCR. For cases with negative CIM test, 30.6% (38/124) was positive and 26.6% (33/124) was negative with multiplex PCR.

**Table 1:** Demographic Characteristics and Clinical Diagnostics of the Patients

Characteristics	*H. pylori *positive	*H. pylori* negative	Total
	53.2% (n = 107)	46.8% (n = 94)	(n = 201)
Mean age (range) (yr)	39.29 (18-72)	41.97 (22-74)	40.5 (18-74)
Sex (female/male)	56/51	59/35	115/86
Diagnosis			
Gastritis	105	88	193
Gastric ulcer	2	6	8

**Table 2:** Diagnostic Accuracy of CLO test, CIM Test and Multiplex PCR

*H. pylori* infection status*		CLO test		CIM		Multiplex PCR	
		Positive	Negative	Positive	Negative	Positive	Negative
Positive (107)		77	30	96	11	107	0
Negative (94)		0	94	23	71	38	56
Total	n	77	124	119	82	145	56
	%	38.3	61.7	59.2	40.8	72.1	27.9

* The *H. pylori* infection status in this study required at least two positive tests of the three tests, comprising of CLO test, CIM test and multiplex PCR.

**Table 3:** Primers Used for Detection and Genotyping *H. pylori*

Primer name	DNA region(s) amplified	Primer sequence (5’-3’)	Amplicon Size(s) (bp)	Reference(s) or source
VAI-F	*vacA* s1/s2	5’-ATGGAAATACAACAAACACAC-3’	259/286	13, 17
VAI-R		5’-CTGCTTGAATGCGCCAAAC-3’		
VAG-F	*vacA *m1/m2	5’-CAATCTGTCCAATCAAGCGAG-3’	567/642	13, 17
VAG-R		5’-GCGTCAAAATAATTCCAAGG-3’		
cagA-F	*cagA*	5’-GATAACAGGCAAGCTTTTGA-3’	349	17
cagA-R		5’-CTGCAAAAGATTGTTTGGCAGA-3’		
IC3-F	SMAD4	5’-CAGCATCCACCAAGTAATCG-3’	200	This study
IC3-R		5’-TCCCCCCAAGTGACTACAC-3’		

## Discussion

*H. pylori *infection has been determined to be a definite cause of gastric cancer (IARC, 1994). Vietnam belongs to the geographic area with high prevalence of *H. pylori *infection (Ngoan le et al., 2008). The ratio of gastric cancer in Vietnamese is the highest one, compared to other ethnicities in South East Asia, for both genders (Kimman et al., 2012). Therefore, accurate *H. pylori *diagnosis is necessary and has a crucial role for successful *H. pylori *eradication. Many methods with different principles could be used to diagnose *H. pylori*, such as CLO test or urea breath test (the activity of urease), PCR (the amplification of conserved genes), stool test (antigen detection), ELISA (antibody detection), serology rapid test (CIM detection), histology (presence of typical bacteria) and culture. Taking advantage of each method could help to minimize noising factors affecting the diagnostic results, especially false negative cases (Lehours, 2018).

In our study, three methods for detecting *H. pylori *were evaluated, including CLO test, CIM test and multiplex PCR. Each method has its own advantages and disadvantages with specific diagnostic value. However, all these tests showed the substantial agreement (0.6 < κ < 0.8) with over 80% accuracy rate when compared to *H. pylori *infection status as defined.

In this study, CLO test showed the highest agreement (κ = 0.706) with 85.07% accuracy rate compared to *H. pylori *infection status even though the positive rate was just 38.3%. The lowest positive rate would mean that many *H. pylori *positive cases would have been ignored. This mostly came from the high negative results of CLO test. Reasons might come from the patients who had used drugs or antibiotics prior to the CLO test (Uotani and Graham, 2015). Moreover, if not used appropriately, CLO test could produce low sensitivity and cause the treatment process to be delayed or foregone. In cases with negative CLO test, adjunctive tests might be required to confirm the *H. pylori *infection status. Conveniently, the biopsies with negative CLO test could be used directly for multiplex PCR without the process of endoscopy carried out again on the patients. CIM also can be used in these cases as backup method. 

All samples with positive CLO test were also positive with multiplex PCR. This proved that the CLO test’s results were true positive even though other urease-producing microorganisms such as *Neisseria flavescens* and *Pseudomonas fluorescens* that can be present in the gastric mucosa may interfere with the detection of *H. pylori *based on the urease activity (Patel et al., 2013; Zeng et al., 2018). While CLO test is the least expensive, the endoscopy procedure is expensive. Therefore, the invasive test in combination with endoscopy cannot be considered as more cost-effective. Moreover, CLO test’s sensitivity was the lowest (71.96%), compared to the two other tests in this study. For all of these, using CLO test should be with high awareness.

Multiplex PCR also demonstrated a substantial agreement rate (κ = 0.611) with 81.09% accuracy rate regarding the *H. pylori *infection status. Multiplex PCR had excellent sensitivity (100%), even though the specificity was the lowest (59.57%). This could be the reason of the high positive rate of this method (72.1%). If the diagnostic process by PCR was done well, the results could be accepted as positive *H. pylori *infection as studies has recently suggested PCR to be a gold standard for *H. pylori *detection (Patel et al., 2014). However, multiplex PCR might be difficult to apply in routine clinical practice because of the need for expensive equipment and carefully trained technicians to avoid false positive results.

Like the other methods, CIM also had the substantial agreement rate (κ = 0.658) with 83.08% accuracy rate. This test seemed to harmonize the results of *H. pylori *detection by the others. For example, multiplex PCR had high positive rate while CLO test had high negative rate of *H. pylori *detection. CIM was in the middle of *H. pylori *detection rate. Moreover, CIM is an easy-to-use and non-invasive method that could be utilized widely with acceptable sensitivity (89.72%) and specificity (75.53%) (Wang et al., 2008). CIM gave the average positive rate (59.2%), which is relatively close to the positive rate of 53.2% as defined by the positive criteria. 

Our study showed that the combination of three methods in *H. pylori *diagnosis helped to increase the positive rate to over 80% in surveyed samples. This rate was also in good agreement with the *H. pylori *infection prevalence in Vietnam (Fock and Ang, 2010; Binh et al., 2017). Therefore, the combination of different methods in *H. pylori *detection is necessary to reflect the accurate diagnostic results and the patients’ accurate *H. pylori *infection status (Krogfelt et al., 2005). 

Besides, UBT is the most investigated and best recommended non-invasive test, according to guidelines, in the context of a “test-and-treat” strategy for diagnosing *H. pylori *infection and should be the reference test for other diagnostic tests to be evaluated. However, most of Vietnamese patients have poor economic situations. They need to have simple, convenient, cost-effective and accurate testing, such as non-invasive test, for example CIM test or gastric endoscopic diagnosis combined with CLO test or multiplex PCR. Moreover, in Vietnam, gastric cancer contains high prevalence, ranking 14th all over the world (GlobalScan, 2018), and rejuvenates. Diagnostic methods for *H. pylori *and gastric lesions detection simultaneously seem to be suitable to the situation. Therefore, patients with epigastric pain are usually advised by clinical doctors to receive endoscopic examination to evaluate possible lesions and to exclude gastric cancer before the treatment. Only using CLO test might cause false negative because of low sensitivity of this test in spite of its high specificity. When combining with PCR, the sensitivity of the *H. pylori *detection will be improved. This suggested that endoscopy with CLO test and multiplex PCR is suitable for Vietnamease patients, especially before eradication regimen. Also in this context, CIM test’s diagnostic value stood between CLO test and multiplex PCR. In addition, it’s convenient because of blood samples for testing and might be applied for children. For above reasons, CIM test might be used as primary diagnostic test before treatment in Vietnam, especially for children and in rural areas where endoscopic combined with multiplex PCR or UBT is not available. In general, there has not been any gold standard method for *H. pylori *diagnosis, and increased accuracy is obtained by using multiple diagnostic tests (Chey et al., 2007; Ansari and Yamaoka, 2018). For Vietnamese patients with gastritis and gastric ulcers, CIM test, CLO test and multiplex PCR could be used for *H. pylori *infection diagnosis with high accuracy as shown in this study; however, in suspected cases, the tests should be employed in combination with the other tests to reduce false-negative results in the clinical settings, especially for CLO test.

Our study had several limitations. Firstly, the patients were only from one hospital in Ho Chi Minh City of the southern Vietnam, though it is one of the biggest hospitals which absorb patients from most provinces around the area. Therefore, these patients did not represent the general Vietnamese population. Secondly, most of the recruits were gastritis patients and some with gastric ulcer, so the results might not be representative of other diseases, such as gastroduodenal ulcer, reflux esophagitis and gastric cancer. Other studies should be performed on these types of diseases to prove the usefulness of applying these tests. Thirdly, the Maastricht V/Florence consensus recommended for assessment of *H. pylori *gastritis, a minimum standard biopsy setting is two biopsies from the antrum (greater and lesser curvature 3 cm proximal to the pyloric region) and two biopsies from the middle of the body; however, in this study just two gastric biopsies were obtained from the antrum for diagnosing *H. pylori*, by CLO test and multiplex PCR. This could produce negative results with CLO test and/or multiplex PCR, but positive with CIM test. In these cases, patients should be advised to take one more test, for example UBT, to make sure *H. pylori *infection status of patients. 

However, the strength of this study was that the three methods of *H. pylori *infection diagnosis with different principles were evaluated, according to the European guidelines for the confirmation of *H. pylori *infection in a patient. Furthermore, in clinical practices, it’s hard to collect many gastric biopsy specimens for the testings, such as CLO test, PCR, and histology at the same time, unless it’s really necessary. This study also showed that using two biopsy samples could give satisfactory results indirectly and specimens used for CLO test could be used for PCR conveniently. The results showed diagnostic values of each method on Vietnamese patients with gastritis and gastric ulcer. These also are two of the most common gastroduodenal disorders. As the Maastricht V/Florence consensus report recommended using non-invasive methods (such as CIM test here) locally validated over endoscopic procedures for the diagnosis of *H. pylori *infection in patients with dyspeptic symptoms (Malfertheiner et al., 2017), this study showed the usefulness of applying CIM test in a Vietnamese population and in our knowledge, similar studies have not been carried out before in the area.

In summary, this study has shown that the combination of different invasive and non-invasive tests can accurately diagnose *H. pylori* infection status. This could in turn enable patients to receive timely and proper treatment. Among the three tests, CIM demonstrated the ability to be used routinely and could be helpful for epidemiological studies of current *H. pylori* infection in areas where endoscopy with CLO test or PCR is not available. In future, similar studies should be conducted in other types of gastroduodenal disorders as well as in comparison of these tests with UBT as a gold standard in other Vietnamese populations.

## References

[B1] Anh NT, Trung TT, Duong HHT (2010). Protocol for the detection of vacA genotypes and cagA gene of Helicobacter pylori from gastric biopsies by a novel multiplex PCR assay. Vietnamese J Gastroenterol.

[B2] Ansari S, Yamaoka Y (2018). Current understanding and management of Helicobacter pylori infection: an updated appraisal. F1000Res.

[B3] Bah A, Saraga E, Armstrong D (1995). Endoscopic features of Helicobacter pylori-related gastritis. Endoscopy.

[B4] Binh TT, Tuan VP, Dung HDQ (2017). Advanced non-cardia gastric cancer and Helicobacter pylori infection in Vietnam. Gut Pathog.

[B5] Chey WD, Wong BC, Gastroenterology PPCotACo (2007). American College of Gastroenterology guideline on the management of Helicobacter pylori infection. Am J Gastroenterol.

[B6] Fock KM, Ang TL (2010). Epidemiology of Helicobacter pylori infection and gastric cancer in Asia. J Gastroenterol Hepatol.

[B7] Hoang TT, Bengtsson C, Phung DC (2005). Seroprevalence of Helicobacter pylori infection in urban and rural Vietnam. Clin Diagn Lab Immunol.

[B8] Hosseini E, Poursina F, de Wiele TV (2012). Helicobacter pylori in Iran: A systematic review on the association of genotypes and gastroduodenal diseases. J Res Med Sci.

[B9] Hung CT, Leung WK, Chan FK (2002). Comparison of two new rapid serology tests for diagnosis of Helicobacter pylori infection in Chinese patients. Dig Liver Dis.

[B11] Kimman M, Norman R, Jan S (2012). The burden of cancer in member countries of the Association of Southeast Asian Nations (ASEAN). Asian Pac J Cancer Prev.

[B12] Krogfelt KA, Lehours P, Megraud F (2005). Diagnosis of Helicobacter pylori Infection. Helicobacter.

[B13] Kumar S, Kumar A, Dixit VK (2008). Direct detection and analysis of vacA genotypes and cagA gene of Helicobacter pylori from gastric biopsies by a novel multiplex polymerase chain reaction assay. Diagn Microbiol Infect Dis.

[B14] Landis JR, Koch GG (1977). An application of hierarchical kappa-type statistics in the assessment of majority agreement among multiple observers. Biometrics.

[B15] Lehours P (2018). Actual diagnosis of Helicobacter pylori infection. Minerva Gastroenterol Dietol.

[B16] Malfertheiner P, Megraud F, O’Morain CA (2017). Management of Helicobacter pylori infection-the Maastricht V/Florence Consensus Report. Gut.

[B17] Ngoan le T, Anh NT, Huong NT (2008). Gastric and colorectal cancer mortality in Viet Nam in the years 2005-2006. Asian Pac J Cancer Prev.

[B18] Nguyen TL, Uchida T, Tsukamoto Y (2010). Helicobacter pylori infection and gastroduodenal diseases in Vietnam: a cross-sectional, hospital-based study. BMC Gastroenterol.

[B19] Park CY, Kwak M, Gutierrez O (2003). Comparison of genotyping Helicobacter pylori directly from biopsy specimens and genotyping from bacterial cultures. J Clin Microbiol.

[B20] Patel SK, Pratap CB, Jain AK (2014). Diagnosis of Helicobacter pylori: what should be the gold standard?. World J Gastroenterol.

[B21] Patel SK, Pratap CB, Verma AK (2013). Pseudomonas fluorescens-like bacteria from the stomach: a microbiological and molecular study. World J Gastroenterol.

[B22] Pellicano R, Ribaldone DG, Fagoonee S (2016). A 2016 panorama of Helicobacter pylori infection: key messages for clinicians. Panminerva Med.

[B23] Schabereiter-Gurtner C, Hirschl AM, Dragosics B (2004). Novel real-time PCR assay for detection of Helicobacter pylori infection and simultaneous clarithromycin susceptibility testing of stool and biopsy specimens. J Clin Microbiol.

[B24] Tongtawee T, Kaewpitoon S, Kaewpitoon N (2016). Diagnosis of Helicobacter pylori infection. Asian Pac J Cancer Prev.

[B25] Trung TT, Anh NT, Loc QH (2013). Helicobacter pylori predictive values by multiplex PCR comparing with CLO test and serology. Ho Chi Minh City J Med.

[B26] Uotani T, Graham DY (2015). Diagnosis of Helicobacter pylori using the rapid urease test. Ann Transl Med.

[B27] Versalovic J (2003). Helicobacter pylor Pathology and diagnostic strategies. Am J Clin Pathol.

[B28] Wang XY, Yang Y, Shi RH (2008). An evaluation of a serologic test with a current infection marker of Helicobacter pylori before and after eradication therapy in Chinese. Helicobacter.

[B29] Zeng B, Sun L, Chen Y (2018). Neisseria flavescens: A Urease-expressing potential pathogen isolated from gastritis patients. Curr Microbiol.

